# Frequency of cataract surgery and its impact on visual function—results from the German Gutenberg Health Study

**DOI:** 10.1007/s00417-020-04770-0

**Published:** 2020-06-08

**Authors:** Alexander K. Schuster, S. Nickels, N. Pfeiffer, I. Schmidtmann, P. S. Wild, T. Münzel, M. E. Beutel, K. J. Lackner, U. Vossmerbaeumer

**Affiliations:** 1 Department of Ophthalmology, University Medical Center of the Johannes Gutenberg-University Mainz, Langenbeckstr. 1, Mainz, 55131 Germany; 2 Institute of Biomedical Statistics, Epidemiology and Informatics, University Medical Center of the Johannes Gutenberg-University Mainz, Mainz, Germany; 3 Preventive Cardiology and Preventive Medicine, Center for Cardiology, University Medical Center of the Johannes Gutenberg-University Mainz, Mainz, Germany; 4 Center for Thrombosis and Hemostasis, University Medical Center of the Johannes Gutenberg-University Mainz, Mainz, Germany; 5 DZHK (German Center for Cardiovascular Research), partner site Rhine-Main, Mainz, Germany; 6 Center for Cardiology, Cardiology I, University Medical Center Mainz, Mainz, Germany; 7 Department of Psychosomatic Medicine and Psychotherapy, University Medical Center Mainz, Mainz, Germany; 8 Institute of Clinical Chemistry and Laboratory Medicine, University Medical Center Mainz, Mainz, Germany

**Keywords:** Epidemiology, Cataract surgery, Pseudophakia, Visual acuity, Quality of life

## Abstract

**Purpose:**

To determine the frequency of cataract surgery in Germany and to evaluate its impact on visual function in an adult population.

**Methods:**

The population-based Gutenberg Health Study was conducted in Germany with its baseline examination between 2007 and 2012 and a 5-year follow-up examiantion. An ophthalmological examination including slit-lamp examination, ocular biometry, and Scheimpflug imaging was carried out. Overall and age-specific frequencies of unilateral and bilateral cataract surgery within 5 years were computed including the 95% confidential intervals [95%-CI]. Association analyses were conducted to determine social and ocular associated factors using multivariable logistic regression analysis. Vision-related quality of life was assessed using NEI VFQ-25.

**Results:**

A total of 10,544 people aged 35 to 74 years were bilateral phakic at baseline and had information on lens status at the 5-year examination. Of these, 168 had unilateral cataract surgery (1.6% [1.4–1.9%]), and 448 had bilateral cataract surgery (4.2% [3.9–4.7%]) in the following 5 years. The frequency of cataract surgery increased with age: 45–54-year-old subjects had twice as often cataract surgery (in at least on eye: OR = 2.32) than at age 35–44 years. The frequency further strongly increases with age (55–64 years: OR = 10.5; 65–74 years: OR = 43.8, *p* < 0.001). Subjects with glaucoma were more likely to have cataract surgery (OR = 2.52, *p* < 0.001). Visual function increased when undergoing bilateral cataract surgery.

**Conclusions:**

The frequency of cataract surgery is low at younger ages and increases up to 26% at age 70–74 years. Persons with glaucoma are more likely to undergo cataract surgery at population-based level in Germany.

**Electronic supplementary material:**

The online version of this article (10.1007/s00417-020-04770-0) contains supplementary material, which is available to authorized users.

## Introduction

Cataract is the leading cause of visual impairment and blindness worldwide, especially in developing countries [1]. In the western countries, cataract surgery including phacoemulsification and implantation of an artificial intraocular lens is the most common procedure to remove the clouded lens and to restore vision loss due to cataract.

While the frequency and outcome of cataract surgery in developing countries is well documented [2–10], the frequency of cataract surgery in industrialized countries and its impact on daily life activities is less investigated [11–14]. Several technological improvements in the last two decades have improved cataract surgery resulting in a relatively safe and common procedure. As part of refractive surgery, even clear lens extractions are nowadays performed [15].

We recently described the prevalence of pseudophakia and aphakia and its impact on vision-related quality of life in the German population [16]. Now, we move forward and analyze the frequency of cataract surgery within 5 years and describe factors leading to higher frequency of cataract surgery. In addition, the impact of cataract surgery on vision-related quality of life is evaluated.

## Materials and methods

The Gutenberg Health Study (GHS) is a prospective, population-based, observational cohort study conducted in the Rhine-Main region in Germany. The sample of 15,010 participants was randomly drawn from local governmental registry offices. The study cohort was equally stratified by sex within each decade of age. More details regarding the study design are described in Höhn et al. [17]. In brief, the baseline examination of the study cohort was carried out between 2007 and 2012 and the 5-year follow-up between 2012 and 2017. The study protocol and study documents were approved by the local ethics committee of the Medical Chamber of Rhineland-Palatinate, Germany (reference no. 837.020.07; original vote: 22.3.2007, latest update: 20.10.2015). According to the tenets of the Declaration of Helsinki, written informed consent was obtained from all participants prior to entering them in the study.

For each participant, a comprehensive ophthalmological examination was conducted, including objective refraction (Humphrey Automated Refractor/Keratometer (HARK) 599, Carl Zeiss Meditec AG, Jena, Germany) and distance-corrected visual acuity, non-contact tonometry (Nidek NT-2000, Nidek Co, Japan) at baseline and follow-up. Slit-lamp examination of the anterior segment and funduscopy was performed at baseline. Ophthalmic conditions (e.g., phakia, pseudophakia and aphakia) were documented using standardized documentation sheets. These variables (aphakia, pseudophakia) were validated using refraction and the ophthalmic medical history. Eye diseases were recorded as self-report for glaucoma, age-related macular degeneration, and corneal disease.

At 5-year follow-up, Scheimpflug imaging, ocular biometry, and fundus photography was conducted. In all eyes with a lens thickness ≤ 2 mm or missing value, Scheimpflug images were evaluated by two trained graders (AKS, PW) for the presence of a crystalline lens (examples are given in supplemental figure 1). In case of deviation (kappa = 0.99 for inter-rater reliability), an experienced cataract and refractive surgeon (UVb) took the decision. In addition, a masked sample of eyes with lens thickness > 2 mm was included.

Vision-related quality of life was assessed using the German version of the National Eye Institute 25-Item Visual Function Questionnaire (NEI VFQ-25) [18, 19]. The questionnaire was self-administered as print-out and Rasch-based analysis was computed to result in measures for visual function scale (VFS) and socio-emotional scale (SES), as described before [20, 21].

Diabetes was diagnosed in those individuals with HbA1c ≥ 6.5%, taking diabetic medication or having been diagnosed by a physician.

Socioeconomic status was defined according to the index used for the German Health Update 2009 (GEDA) and ranged from 3 to 21 [22]. The type of health insurance (private vs. statutory) was questioned.

### Study population

All subjects with bilateral phakia at baseline examination were included for data analysis. Individuals with unilateral phakia were included in a separate analysis sample.

### Data and statistical analysis

All variables were first tested for normal distribution. Medians, interquartile ranges, minimums, and maximums were calculated for all primary and secondary variables. For variables that were normally distributed, means and standard deviations were computed as well.

The primary outcome was lens status at 5-year follow-up examination. Secondary outcome was monocular distant-corrected visual acuity, and vision-related quality of life as visual functioning scale and socio-emotional scale.

First, age-specific frequency of cataract surgery was calculated including 95% confidence intervals. Associated parameters were evaluated using multivariable logistic regression models. Models were conducted to compare (1) people with unilateral cataract surgery to those without cataract surgery, (2) people with bilateral cataract surgery to those without cataract surgery, and (3) people with any cataract surgery. The included covariates were age (in decades), sex, socioeconomic status, diabetes, type of health insurance, self-reported age-related macular degeneration, glaucoma, and corneal disease. These variables were chosen, as aging and diabetes are well-known risk factor for cataract, while the influence of social factors such as socioeconomic status, sex, and type of health insurance on cataract surgery might differ between countries. The different eye diseases were included to investigate whether glaucoma was related to a higher frequency of cataract surgery in a population-based study setup, while self-report of age-related macular degeneration and corneal disease was supposed to not have any effect, although these subjects may more frequently consult an ophthalmologist.

An intra-individual comparison (baseline to follow-up examination) was performed to evaluate differences in refraction (sphere, astigmatism), visual acuity, and vision-related quality of life.

Self-reported cataract surgery within the last 5 years at follow-up was compared to objective measure of cataract surgery and kappa-statistics were computed.

This study was performed as an explorative study to analyze the frequency of cataract surgery and the factors associated with this condition. All *p* values should be regarded as continuous parameters that reflect the level of evidence and are therefore reported exactly. The data were processed using statistical analysis software (R version 3.5.2 [2018-12-20]).

## Results

A total of 13,993 study subjects had bilateral phakia at baseline examination, and thereof, 11,669 (83.4%) re-attended the 5-year follow-up examination. Of them, bilateral lens status data were missing in 1125 people (9.6%) at 5-year follow-up leading to 10,544 subjects included in this analysis. Item-non-responder analysis showed that participants with missing information were older, had a lower socioeconomic status, and had more systemic comorbidities (supplemental table 1), while they were similar in eye parameters. In a second analysis sample, those with one eye phakic and the fellow eye pseudophakic at baseline examination and lens status measurement at 5-year follow-up examination were included (*n* = 139) (Table 1).

**Table 1:** Baseline characteristics of the analysis sample. Study participants from the Gutenberg Health Study with bilateral/unilateral phakia at baseline (2007–2012) and lens status at 5-year follow-examination (2012–2017)

Baseline data	Bilateral phakia at baseline	Unilateral phakia at baseline
Number (*n*)	10,544	139
Sex (women)	5129 (48.6%)	64 (46.0%)
Age (years)	53.84 ± 10.67	64.73 ± 7.62
Socioeconomic status	13.3 ± 4.4	13.1 ± 4.9
Body mass index (kg/m^2^)	27.1 ± 4.8	27.7 ± 4.3
Diabetes (yes)	754 (7.2%)	22 (15.8%)
Arterial hypertension (yes)	4927 (46.7%)	87 (62.6%)
Type of health insurance		
Private (yes, otherwise statutory)	271 (3.3%)	2 (2.0%)
Ophthalmic parameters		
Visual acuity OD (median [IQR])	0.00 [0.00, 0.10]	0.22 [0.00, 0.22]^a^
Visual acuity OS (median [IQR])	0.00 [0.00, 0.10]	0.22 [0.00, 0.30]
Spherical equivalent (diopter) OD	− 0.47 ± 2.51	− 0.86 ± 2.61^b^
Spherical equivalent (diopter) OS	− 0.46 ± 2.52	− 0.65 ± 2.43
Intraocular pressure (mmHg) OD	14.10 ± 2.81	14.26 ± 3.12^c^
Intraocular pressure (mmHg) OS	14.25 ± 2.86	14.18 ± 2.79
Eye diseases (self-reported)		
Glaucoma	179 (1.7%)	13 (9.4%)
Diabetic retinopathy	31 (0.3%)	3 (2.2%)
Age-related macular degeneration	31 (0.3%)	4 (2.9%)
Corneal disease	183 (1.7%)	7 (5.0%)

^a^With respect to phakic/pseudophakic eyes: 0.18 ± 0.20/0.25 ± 0.39

^b^With respect to phakic/pseudophakic eyes: − 0.90 ± 3.35/− 0.68 ± 2.12

^c^With respect to phakic/pseudophakic eyes: 14.22 ± 2.98/13.85 ± 3.04

**Fig. 1:**
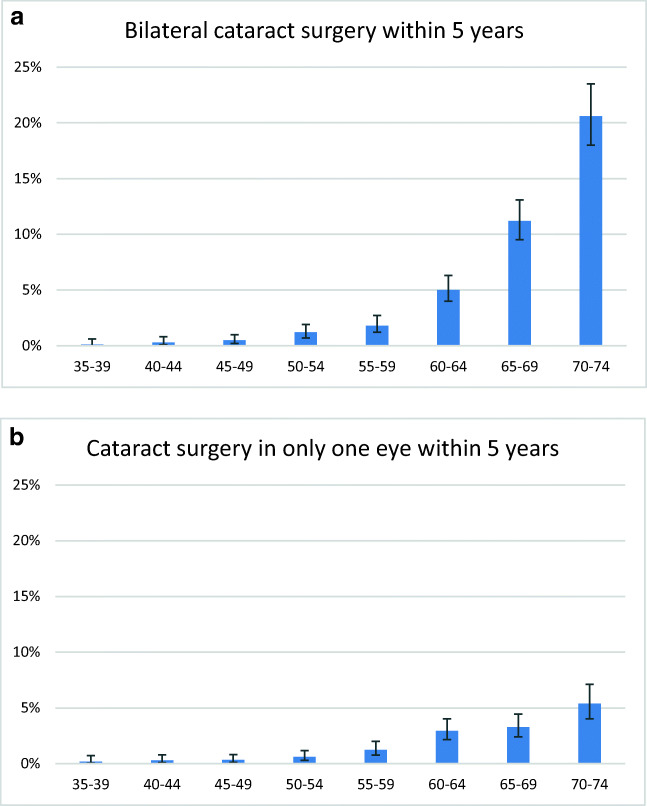
Age-specific frequencies and 95% confidential intervals for cataract surgery within the last 5 years. Data from the population-based German Gutenberg Health Study (2007–2017) analyzing study participants being bilateral phakic at baseline examination. **a** bilateral cataract surgery. **b** unilateral cataract surgery

Four hundred forty-eight of 10,544 people (4.2%; 95% confidence interval [3.9–4.7%]) underwent bilateral cataract surgery, while 168 (1.6% [1.4–1.9%]) had unilateral cataract surgery. For both, an increase with age was observed (Fig. 1). While at age 35–39 years only 0.28% had cataract surgery in the following 5 years in at least one eye, this frequency increased up to 26.0% at age 70–74 years.

The agreement between self-reported cataract surgery during the recent 5 years and an observed change in the lens status was 99.2% (Cohen’s Kappa = 0.93), when analyzing the change in at least one eye.

Logistic regression analyses showed that age, diabetes, and glaucoma were independently associated with undergoing cataract surgery in the next 5 years (Table 2). Neither sex, nor socioeconomic status, health insurance status, and corneal disease did have an impact on cataract surgery in the following 5 years in one or both eyes.

**Table 2:** Association analysis of factors leading to cataract surgery within 5 years. Data from the German Gutenberg Health Study (2007–17) including bilateral phakic subjects at baseline. Multivariable logistic regression analysis was computed

	Unilateral cataract surgery		Bilateral cataract surgery		Any cataract surgery	
	OR (95% CI)	*p*	OR (95% CI)	*p*	OR (95% CI)	*p*
Age:		< 0.001		< 0.001		< 0.001
35–44 years	Reference		Reference		Reference	
45–54 years	1.54 (0.57; 4.18)		3.48 (1.17; 10.37)		2.32 (1.12; 4.78)	
55–64 years	6.93 (2.93; 16.4)		15.7 (5.73; 43.2)		10.5 (5.50; 20.2)	
65–74 years	14.2 (6.08; 33.3)		79.5 (29.4; 216)		43.8 (23.2; 83.0)	
Sex (female)	1.09 (0.76; 1.57)	0.64	1.09 (0.87; 1.38)	0.44	1.11 (0.90; 1.35)	0.33
Socioeconomic status	1.02 (0.97; 1.06)	0.49	0.98 (0.95; 1.01)	0.18	0.99 (0.97; 1.02)	0.45
Diabetes (yes)	1.06 (0.62; 1.83)	0.82	1.44 (1.06; 1.96)	0.019	1.36 (1.03; 1.79)	0.031
Health insurance (private)	0.80 (0.25; 2.57)	0.70	1.40 (0.74; 2.64)	0.31	1.22 (0.69; 2.15)	0.50
Glaucoma (yes)	1.72 (0.74; 4.02)	0.24	2.56 (1.59; 4.14)	<0.001	2.52 (1.62; 3.93)	<0.001
AMD (yes)	–	–	2.79 (1.04; 7.49)	0.059	2.04 (0.76; 5.45)	0.18
Corneal disease (yes)	0.61 (0.15; 2.51)	0.46	1.06 (0.55; 2.05)	0.87	0.94 (0.69; 2.15)	0.84

**Fig. 2:**
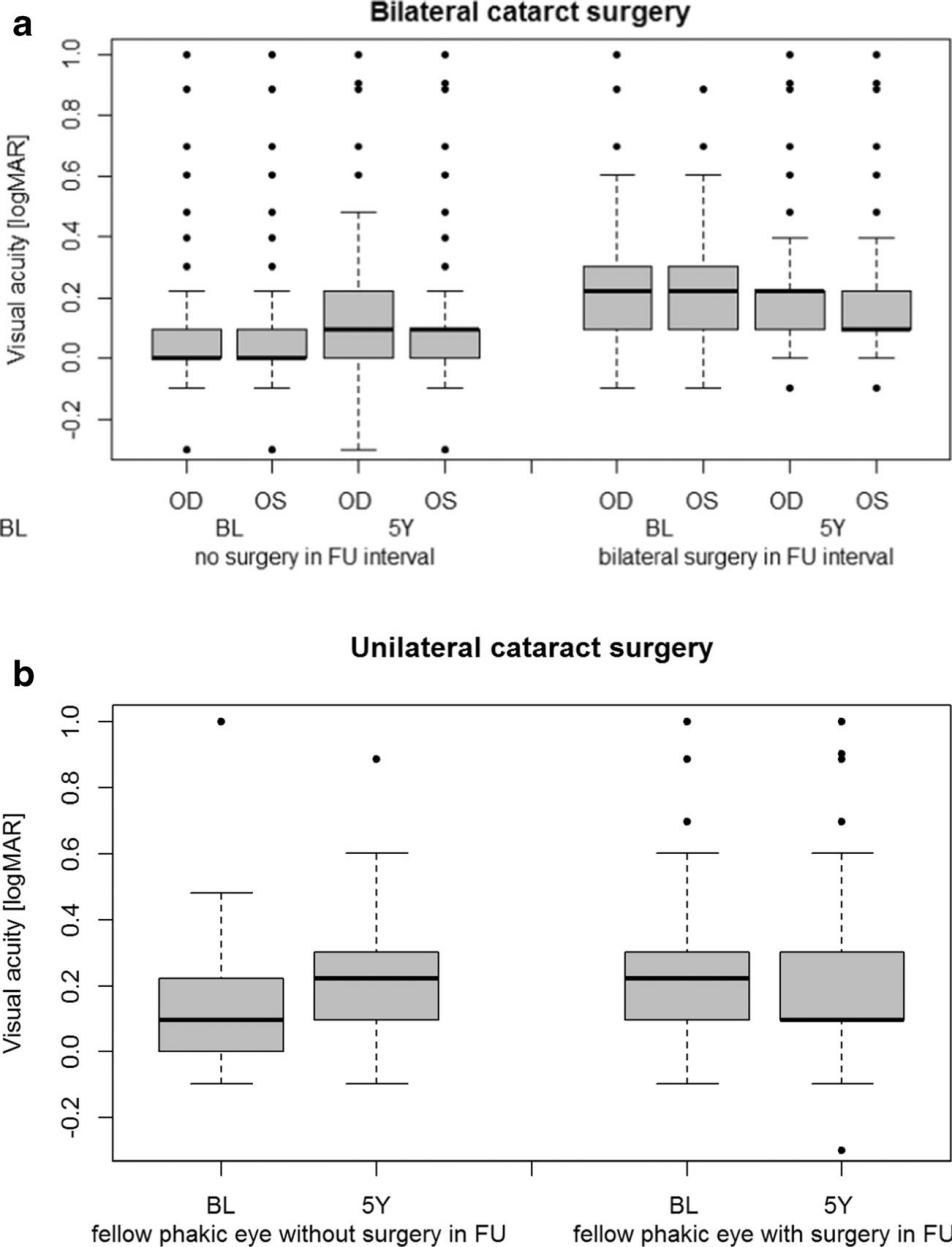
Distant-corrected visual acuity decreases in subjects without cataract surgery over 5 years, while increases in those with bilateral cataract surgery. Data at baseline and 5-year follow-up examination in eyes of participants with **a** both eyes without cataract surgery at baseline and bilateral cataract surgery during 5-year follow-up interval and **b** both eyes without cataract surgery at baseline and unilateral cataract surgery during 5-year follow-up interval. Data from the population-based German Gutenberg Health Study (2007–2017) are presented in box-plots (gray boxes: interquartile range (IQR); black line: median). OD: right eyes; OS: left eyes

Distance-corrected visual acuity decreased by 0.10 logMAR (median) over 5 years in eyes not undergoing cataract surgery to 0.10 logMAR in right eyes and 0.10 in left eyes. Eyes that underwent bilateral cataract surgery had baseline logMAR of 0.22 in both eyes, and 0.22 in right eyes and 0.10 in left eyes at 5-year follow-up after cataract surgery (Fig. 2a). In participants with unilateral surgery, the visual acuity of the treated eye increased from 0.22 to 0.10, while the untreated eye decreased from 0.10 to 0.22 (Fig. 2b).

For those with one eye phakic and the other pseudophakic status (*n* = 139) at baseline, 44.6% [36.3–53.3%] had cataract surgery in the fellow eye within the following 5 years. Visual acuity remained stable in fellow pseudophakic eyes over time, while decreased in eyes without cataract surgery and increased in eyes with cataract surgery over time (supplemental figure 2).

**Fig. 3:**
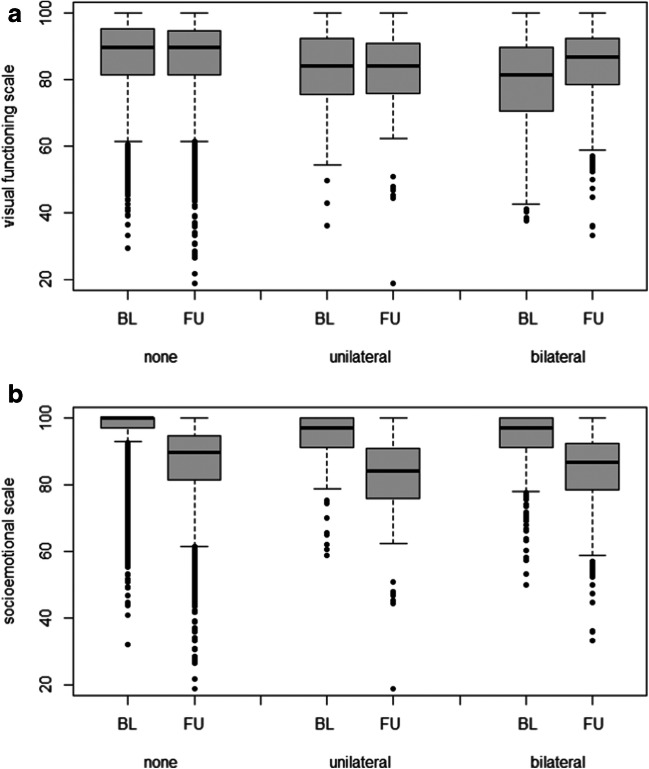
Vision-related Quality of Life increases in the visual functioning scale (**a**) after bilateral cataract surgery, but not in the socio-emotional scale (**b**). Data from the population-based German Gutenberg Health Study (2007–2017) at baseline (BL) and 5-year follow-up interval (FU), based on Rasch-transformed NEI VFQ-25 measurements, are presented in box-plots (gray boxes: interquartile range (IQR); black line: median)

The change in vision-related quality of life in terms of the visual functioning scale score (VFS) of NEI VFQ-25 is similar between participants with no cataract surgery (*n* = 9928) and those with unilateral cataract surgery (*n* = 168). In participants with bilateral cataract surgery (*n* = 448), the median VFS is 5.4 [IQR − 2.8; 13.7] points higher at follow-up than at baseline (Fig. 3). The median socio-emotional scale score decreased in all participants, but the decrease is highest in participants with unilateral cataract surgery and lowest in participants with bilateral cataract surgery (Fig. 3).

## Discussion

For the first time, we report the frequency of cataract surgery in Germany in a population-based study. We found that 4.2% of the 35- to 74-year-old population has bilateral cataract surgery and 1.6% unilateral cataract surgery during an observation interval of 5 years. These people are more likely to be older and to suffer from diabetes. Sex or health insurance status is not associated with cataract surgery in Germany.

There is growing evidence that the prevalence of cataract and its management is linked to socioeconomic factors [23]. Prevalence of age-related cataract is related to lower socioeconomic status in the USA [24] and India [25]. In the Singapore Malay Eye Study, a relationship between indicators of socioeconomic status, namely education level and income, was associated with nuclear sclerotic cataract prevalence [26].

Cataract surgery is reported to be a highly cost-effective treatment for a preventable cause of visual impairment in first and second eyes [27, 28]. Nevertheless, barriers to cataract surgery have been identified, not only in developing countries but also in the USA [29–32]. In the Los Angeles Latino Eye Study, low socioeconomic status was associated with an unmet need for visually significant cataract surgery [29]. We therefore investigated whether socioeconomic status was associated with cataract surgery in Germany as well. Our data showed that undergoing cataract surgery was independent of socioeconomic status, which is not surprising in a country having an obligate health insurance system and covering almost the total population. Costs of cataract surgery with monofocal intraocular lens implantation are reimbursed in both the private and statutory healthcare system. Nevertheless, there are additional private costs for patients undergoing cataract surgery such as measuring the dimensions of the eye with optical biometry for IOL power calculation or when receiving a customized IOL. Unfortunately, our data did not allow to further investigate these aspects.

Cataract surgery is part of the treatment cascade in both open-angle and angle-closure glaucoma. Seol et al. reported reduction in intraocular pressure and less intraocular pressure spikes in normal subjects and normal-tension glaucoma [33]. In primary angle-closure glaucoma, Azuara-Blanco et al. showed that early lens extraction has greater efficacy and is more cost-effective than peripheral laser iridotomy [34]. This was also found in our data: subjects suffering from glaucoma at baseline were more likely to undergo cataract surgery within the following 5 years.

Diabetes and impaired fasting glucose level is a known risk factor for cataract incidence in several population-based studies [35–37]. In Germany, there is a national guideline on diabetic retinopathy screening that incorporates a slit-lamp examination of the eye at least every 1–2 years [38]. Consequently, opacification of the lens will be observed and necessary treatment will be initiated. This association was also present in our analysis: persons suffering from diabetes showed a 1.4-fold higher frequency of cataract surgery independently from aging. Our risk estimates for undergoing cataract surgery were slightly lower than those from a previous multi-ethnic Asian population from Singapore [39], which might be due to differences in diabetic treatment regime and in blood sugar control between the two countries.

In terms of vision-related quality of life, participants who received bilateral cataract surgery benefited the most. This is in line with previous results from the GHS, where we found similar VRQoL in bilateral phakic and pseudophakic subjects, but 6-point lower VRQoL in monolateral pseudophakic [16]. For cataract surgery, multiple studies reported an increase of VRQoL in subjects after cataract surgery of the second eye [40–42]. This is in line with the growing body of evidence that both eyes have a considerable impact on VRQoL, and not only the better-seeing eye [43–45].

There are methodological limitations of this study: first, the lens status was determined by slit-lamp examination at baseline using a neutral pupil. At follow-up examination, lens status was evaluated with LenStar measures and analyzing Scheimpflug images, showing high reliability measures. This might have an impact on our estimates; nevertheless, both methods are held as valid methods for determination of lens status. As our study was solely conducted in Germany, the healthcare system may have an influence on the supply with cataract surgery and cannot be directly generalized to other countries. In addition, our study population was 35 to 74 years old at baseline; therefore, frequencies of cataract surgery within the next 5 years are only valid for this age range. In a population-based study, response to study invitation might be a limitation. In our study, the initial response was 55.5% (effective recruitment efficacy proportion) and the response for the 5-year follow-up examination was 83.4% for the analyzed study sample. As there is no information about eye health in non-responders, we are not able to determine whether people with eye diseases might have less likely participated. Regarding differences in response at follow-up examination, male subjects at younger age and female subjects at older age were less likely to take part.

In summary, the frequency of cataract surgery is low at younger ages and increases up to 26% at age 70–74 years. Persons with glaucoma are more likely to undergo cataract surgery at population-based level in Germany, as are those suffering from diabetes.

## Electronic supplementary material

Supplemental Table 1 (DOCX 1516 kb)

Supplemental Figure 1 (DOCX 1496 kb)

Supplemental Figure 2 (DOCX 45 kb)
