# Supplementary material for: Frequency of cataract surgery and its impact on visual function—results from the German Gutenberg Health Study
Source: Graefes Arch Clin Exp Ophthalmol. 2020 Jun 8;258(10):2223–31. doi: 10.1007/s00417-020-04770-0 (PMC7550321; doi:10.1007/s00417-020-04770-0)

**Supplemental figure 1:** Example of a Scheimpflug image of a phakic eye (a) and a pseudophakic eye (b).

a)


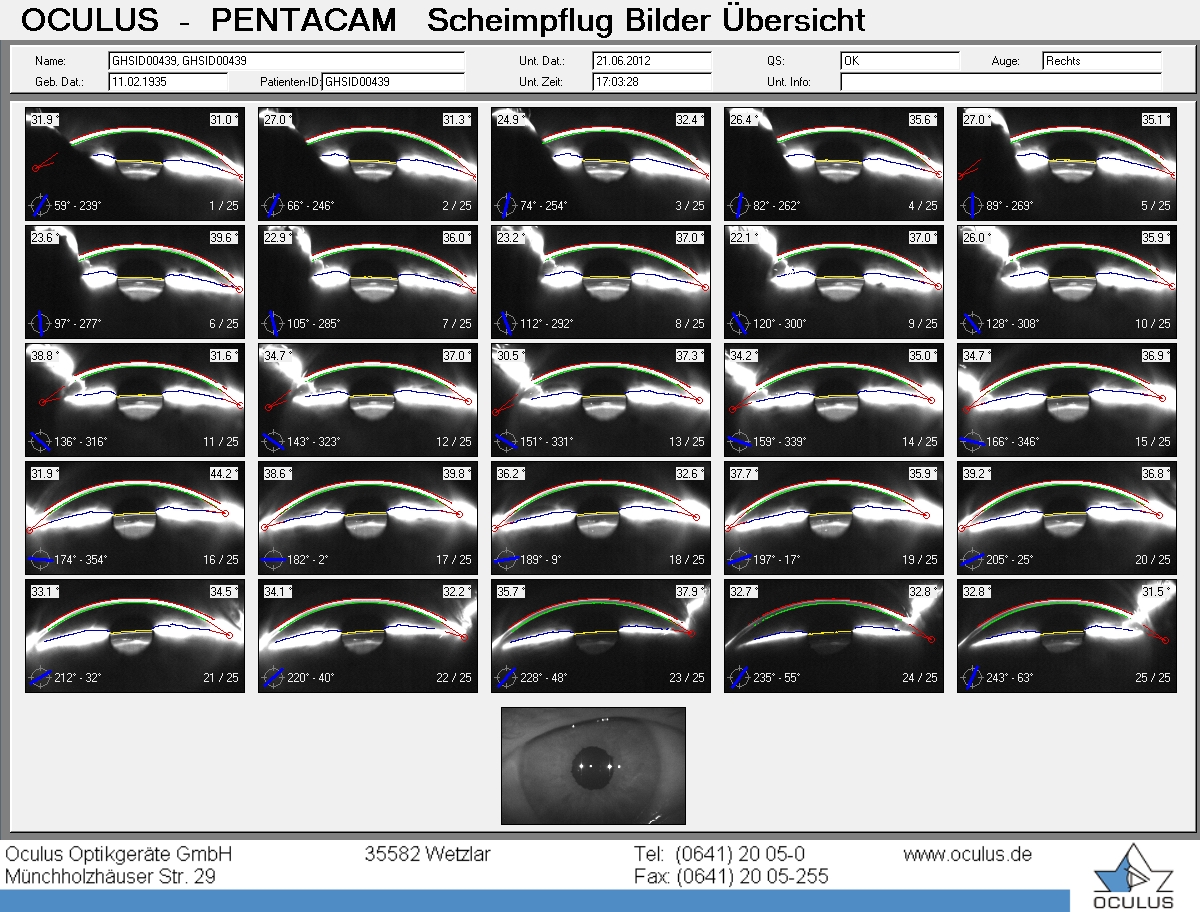


b)


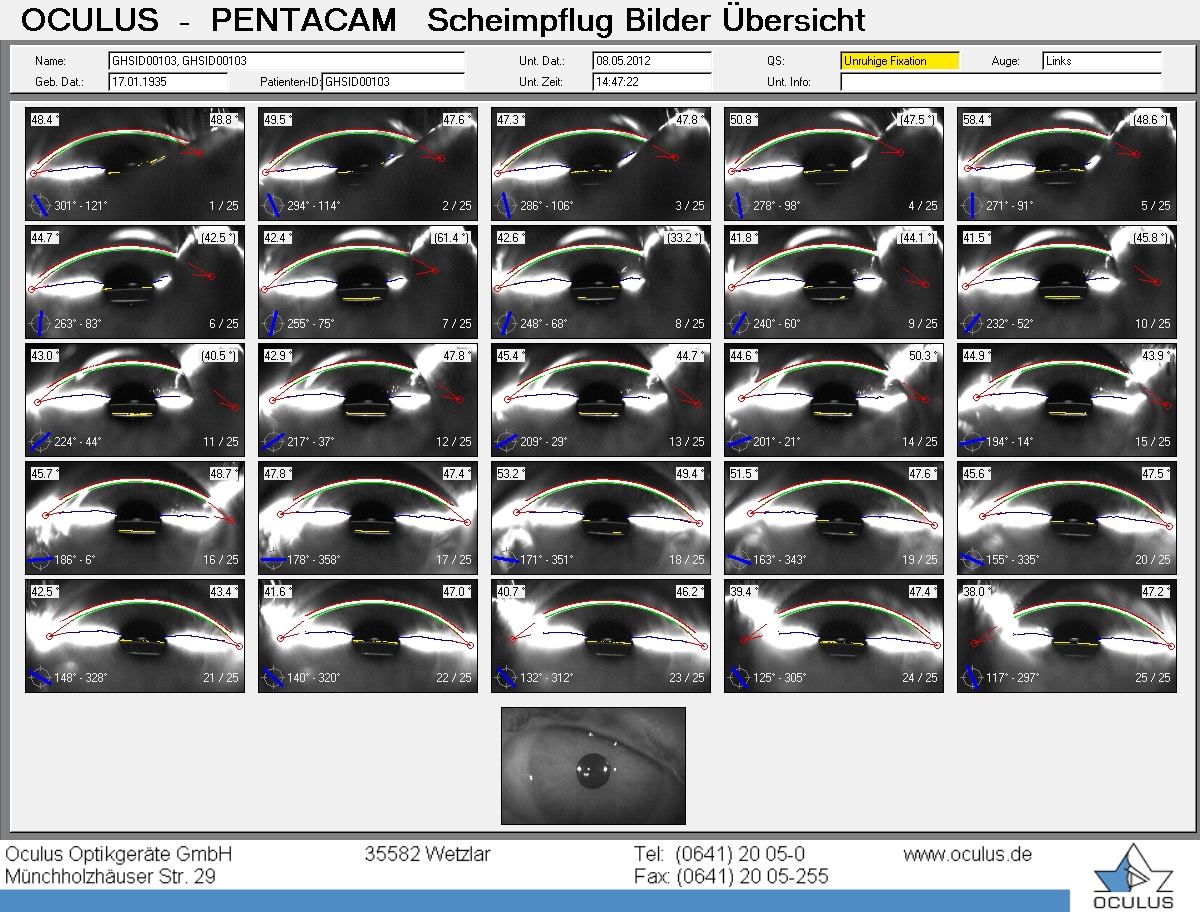


**Supplemental figure 2:** Visual acuity at baseline and 5-year follow-up examination in eyes of participants with one phakic and one pseudophakic eye at baseline (BL) and cataract surgery of the fellow eye during 5-year follow-up interval (5Y). Data from the population-based German Gutenberg Health Study (2007-17) are presented in box-plots (gray boxes: interquartile range (IQR); black line: median; whiskers: lowest data point still within 1.5 (IQR) of the lower quartile, and the highest data point still within 1.5 IQR of the upper quartile; dots: outliers).


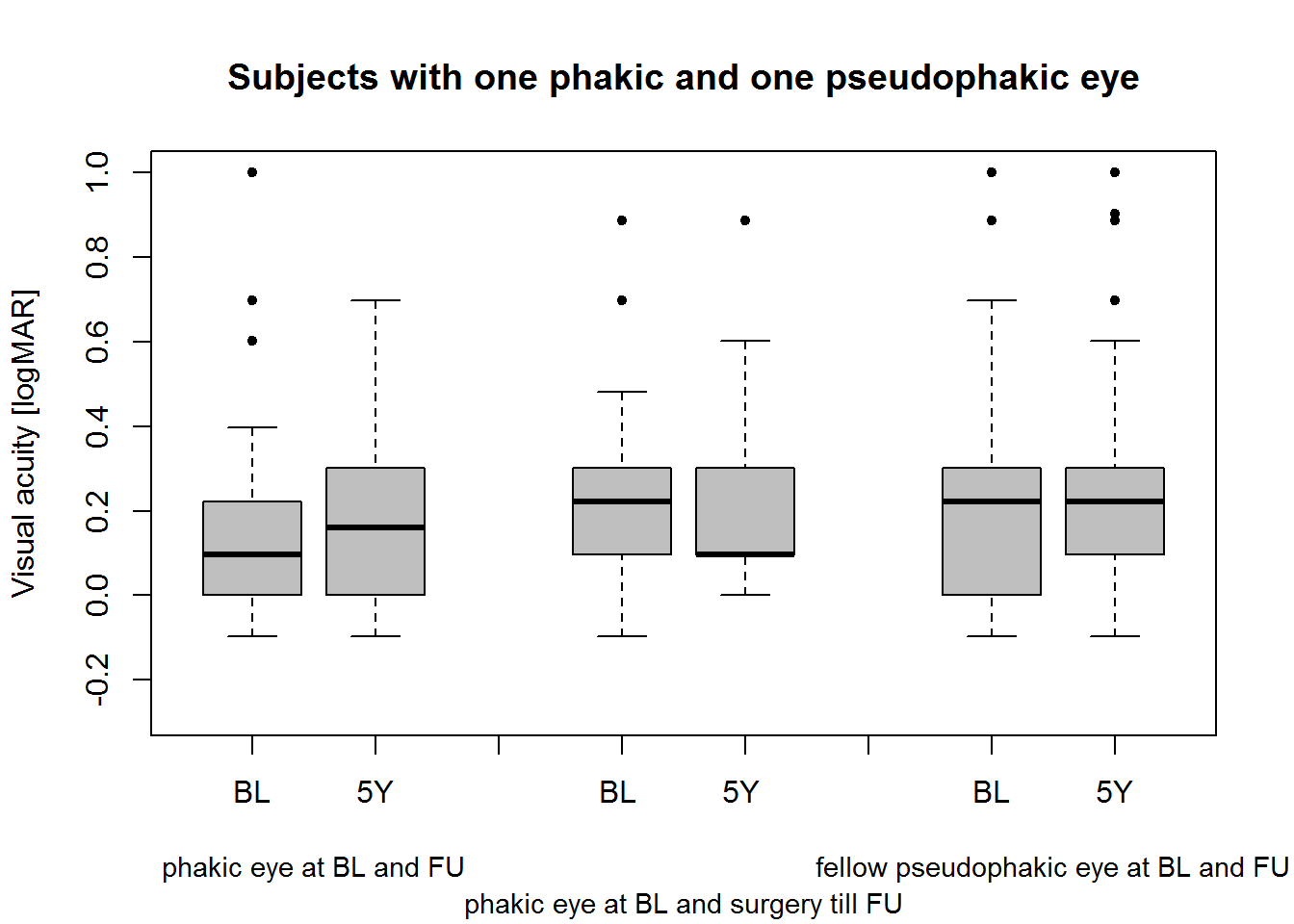

Supplement: Supplementary file 1 — (DOCX 1516 kb) [file 417_2020_4770_MOESM1_ESM.docx]
