# Supplementary figures and images for: Frequency of cataract surgery and its impact on visual function—results from the German Gutenberg Health Study
Source: Graefes Arch Clin Exp Ophthalmol. 2020 Jun 8;258(10):2223–31. doi: 10.1007/s00417-020-04770-0 (PMC7550321; doi:10.1007/s00417-020-04770-0)

**Supplemental figure 1:** Example of a Scheimpflug image of a phakic eye (a) and a pseudophakic eye (b).

a)


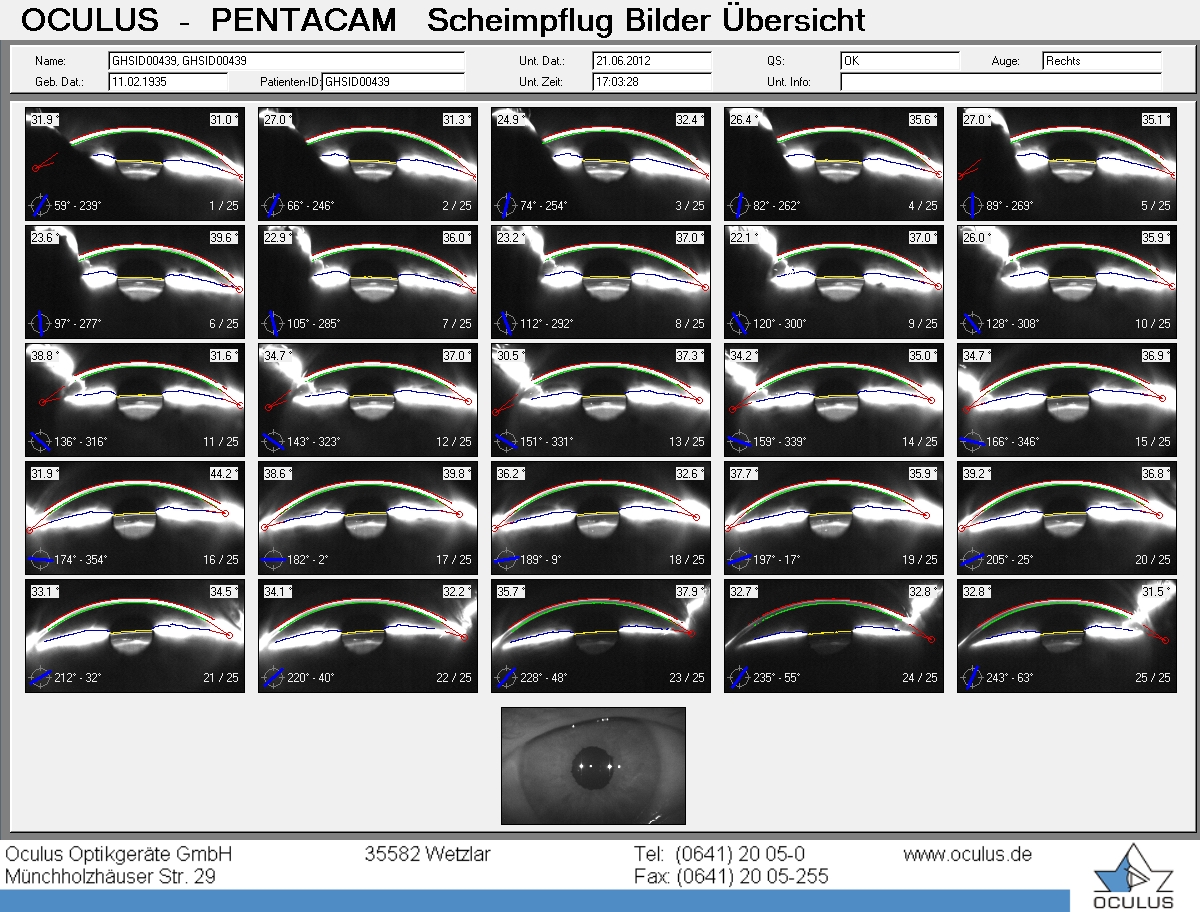


b)


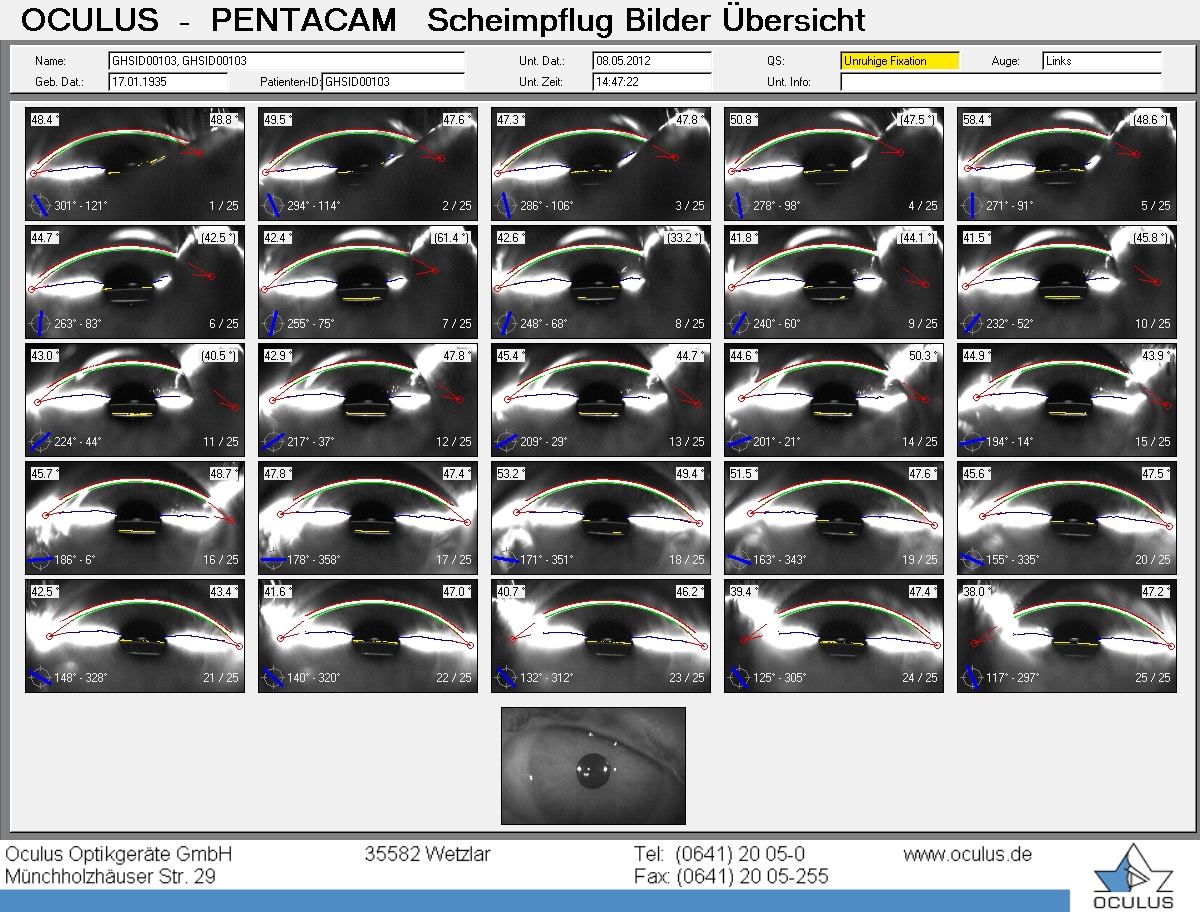

Supplement: Supplementary file 2 — (DOCX 1496 kb) [file 417_2020_4770_MOESM2_ESM.docx]
